# Biochemical, Enzymatic, and Computational Characterization of Recurrent Somatic Mutations of the Human Protein Tyrosine Phosphatase PTP1B in Primary Mediastinal B Cell Lymphoma

**DOI:** 10.3390/ijms23137060

**Published:** 2022-06-24

**Authors:** Rongxing Liu, Yujie Sun, Jérémy Berthelet, Linh-Chi Bui, Ximing Xu, Mireille Viguier, Jean-Marie Dupret, Frédérique Deshayes, Fernando Rodrigues Lima

**Affiliations:** 1 Université Paris Cité, CNRS, Unité de Biologie Fonctionnelle et Adaptative, F-75013 Paris, France; 1173942453lrx@gmail.com (R.L.); linh-chi.bui@u-paris.fr (L.-C.B.); mireille.viguier@u-paris.fr (M.V.); jean-marie.dupret@u-paris.fr (J.-M.D.); frederique.deshayes@u-paris.fr (F.D.); 2 School of Medicine and Pharmacy, Ocean University of China, Qingdao 266071, China; sunyujie@stu.ouc.edu.cn (Y.S.); xmngxu@gmail.com (X.X.); 3 Université Paris Cité, CNRS, Centre d’Epigénétique et Destin Cellulaire, F-75013 Paris, France; berthelet.jeremy1@gmail.com

**Keywords:** lymphoma, protein tyrosine phosphatase, PTP1B, oncogenic mutations, enzyme activity

## Abstract

Human protein tyrosine phosphatase 1B (PTP1B) is a ubiquitous non-receptor tyrosine phosphatase that serves as a major negative regulator of tyrosine phosphorylation cascades of metabolic and oncogenic importance such as the insulin, epidermal growth factor receptor (EGFR), and JAK/STAT pathways. Increasing evidence point to a key role of PTP1B-dependent signaling in cancer. Interestingly, genetic defects in PTP1B have been found in different human malignancies. Notably, recurrent somatic mutations and splice variants of PTP1B were identified in human B cell and Hodgkin lymphomas. In this work, we analyzed the molecular and functional levels of three PTP1B mutations identified in primary mediastinal B cell lymphoma (PMBCL) patients and located in the WPD-loop (V184D), P-loop (R221G), and Q-loop (G259V). Using biochemical, enzymatic, and molecular dynamics approaches, we show that these mutations lead to PTP1B mutants with extremely low intrinsic tyrosine phosphatase activity that display alterations in overall protein stability and in the flexibility of the active site loops of the enzyme. This is in agreement with the key role of the active site loop regions, which are preorganized to interact with the substrate and to enable catalysis. Our study provides molecular and enzymatic evidence for the loss of protein tyrosine phosphatase activity of PTP1B active-site loop mutants identified in human lymphoma.

## 1. Introduction

Primary mediastinal B cell lymphoma (PMBCL) is a rare subtype of aggressive B cell lymphoma that is putatively derived from thymic B cells [1]. Although PMBCL is considered distinct from other B cell non-Hodgkin lymphoma subtypes, it has features that overlap with Hodgkin lymphoma [1]. The malignant cells express B cell markers (CD19, CD20, CD22) and B cell transcription factors such as PAX5, OCT2, and BCL6 [2]. The pathobiology of PMBCL is known to rely on the alteration of different molecular pathways. In particular, different cell signaling cascades such as JAK/STAT and nuclear factor-κB (NF-κB) pathways have been shown to be dysregulated in PMBCL, leading to increased protein tyrosine phosphorylation [3,4,5]. Recently, recurrent mutations in the *PTPN1* gene encoding the human protein tyrosine phosphatase 1B (PTP1B) were reported in PMBCL and Hodgkin lymphoma, thus supporting previous evidence of the involvement of the PTP1B enzyme in lymphomagenesis [6,7]. In addition, an inactive splice variant of PTP1B has been identified in classical Hodgkin lymphoma cells and shown to increase tyrosine phosphorylation signaling [8]. Further studies in mice models have also reported that a lack of PTP1B induces myeloproliferative neoplasm and can lead to the development of acute leukemia [9,10].

PTP1B is a ubiquitous non-receptor tyrosine phosphatase that serves as a major negative regulator of tyrosine phosphorylation cascades of metabolic importance such as insulin or leptin signaling [11,12]. In addition to its metabolic impact, mounting evidence points to a critical role of PTP1B in cancer, serving as a tumor suppressor or promoter depending on the context [13]. In particular, PTP1B plays a pivotal role in the modulation of relevant oncogenic signaling pathways such as the EGFR and the JAK/STAT cascades through tyrosine dephosphorylation of several protein effectors [13,14,15,16,17]. Interestingly, genetic defects impacting these pathways and/or aberrant signaling are known in lymphoma [18,19,20].

All PTPs catalyze the cleavage and hydrolysis of phosphotyrosine in their protein targets. PTP1B shares a common catalytic mechanism based on a nucleophilic cysteine and other important invariant residues that are located in a number of highly conserved adjacent loops that form the active site (such as the pTyr-loop, WPD-loop, P-loop, and Q-loop) [21]. The structure, flexibility, and dynamics of these active site loops (notably the WPD-loop, residues 177–188) but also of adjacent residues are key for catalysis and modulation of PTP1B activity [22,23,24]. In addition, it is now known that there is also a regulatory network (allosteric network) of different residues throughout the enzyme structure (such as Arg56, Ala69, Tyr153 or Asn193) that contribute to PTP1B activity, notably by impacting the motion of the WPD-loop [22,23,25].

As stated above, recurrent somatic mutations of human PTP1B in PMBCL have been recently reported. While these mutations occurred throughout the PTP1B sequence, the majority were nonetheless found in the catalytic domain of the enzyme (spanning residues 1–321) [7]. Analysis of the tyrosine phosphatase activity in cell lysates of these mutants indicated that certain mutations can lead to PTP1B variants with significantly decreased activity whereas other mutations have little impact [7]. Interestingly, three point mutations were found within the WPD-loop (V184D), P-loop (R221G), and Q-loop (G259V) in PMBCL patients. Although the V184D mutation has been shown to reduce the overall tyrosine phosphatase activity of cell lysates overexpressing the PTP1B V184D mutant, the molecular and enzymatic impacts of these three loop mutations on human PTP1B were not addressed [7]. In this manuscript, we analyzed, at the molecular and enzymatic levels, three PTP1B mutations identified in PMBCL patients and located in the WPD-loop (V184D), the P-loop (R221G), and the Q-loop (G259V) of the enzyme [7,21]. We found that recombinantly expressed and purified PTP1B mutants displayed extremely low phosphatase activity. In line with this, the expression of the three PTP1B mutants in cells also led to enzymes with very low intrinsic tyrosine phosphatase activity and a concomitant increase in the tyrosine phosphorylation of EGFR, a known PTP1B substrate. Further biochemical and molecular dynamics approaches indicated that these PTP1B mutations impact overall protein stability, protein dynamics, and the flexibility of the active site PTP1B loops, notably the WPD-loop, P-loop, and Q-loop. Our study provides molecular and enzymatic evidence that the PTP1B active-site loop mutants identified in human lymphoma are strongly impaired in their tyrosine phosphatase activity, thus contributing to aberrant cell signaling.

## 2. Results and Discussion

**Figure 1:**
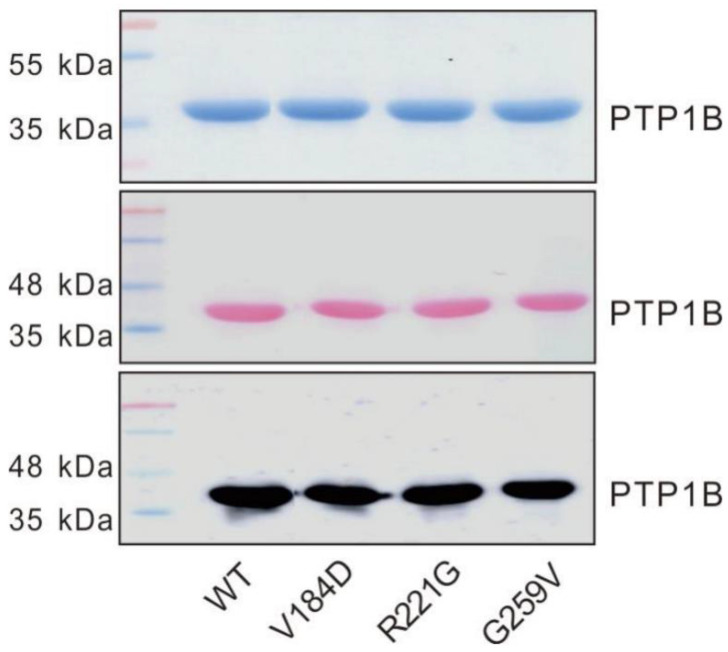
Purification of the PTP1B proteins in the WT and mutants. These PTP1B proteins were separated by SDS-PAGE post-purification and stained using Coomassie dye or further transferred onto nitrocellulose membrane for PTP1B immunodetection following Ponceau staining.

The catalytic domain (residues 1–321) of human WT PTP1B and the V184D, R221G, and G259V PTP1B mutants were expressed in *E. coli* and purified by immobilized metal affinity chromatography (IMAC). This domain was used as it is more amenable to heterologous expression in bacteria and to purification. In addition, the PTP1B catalytic domain has been used in most studies devoted to understanding the catalytic mechanism, the structure, and/or the regulation of PTP1B activity, notably through the use of purified PTP1B mutants [22,23,24,25,26,27]. As shown in Figure 1, the WT and mutant forms of human PTP1B were readily expressed and purified, indicating that the mutations had no gross effects on the expression of the proteins.

**Figure 2:**
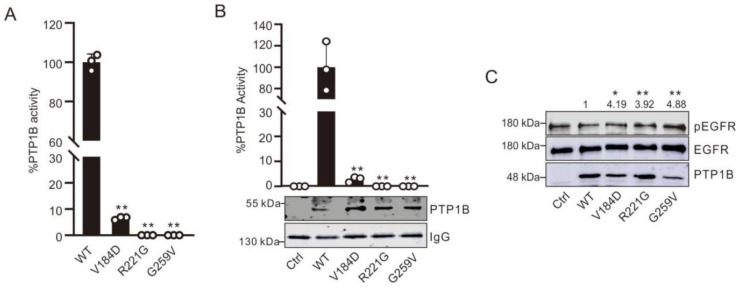
In vitro and in cellulo determination of the PTP1B activity in the WT and mutants. (**A**) PTP1B enzyme (5 nM) activity in the WT or mutants was assessed by RP-UFLC using a pSTAT1 fluorescent peptide. Data are presented as the mean of three independent experiments ± SD. ** *p*-value < 0.01 (compared to WT PTP1B). (**B**) HEK 293T cells transfected with the WT’s or mutants’ flag-PTP1B plasmids were lysed, and PTP1B exogenous proteins were immunoprecipitated and split into two parts: one part was used to assess immunoprecipitated PTP1B residual activity by RP-UFLC using a pSTAT1 fluorescent peptide as described before [28], and the second part was separated by SDS-PAGE and further proceeded for PTP1B immunodetection. Data are presented as the mean of three independent experiments ± SD. * *p*-value < 0.05, ** *p*-value < 0.01 (compared to WT PTP1B). The membrane is representative of three independent experiments. (**C**) COS7 cells transfected with the WT’s or mutants’ flag-PTP1B plasmids were lysed, and samples were separated by SDS-PAGE for tyrosine phosphorylation immunodetection as described by Flint et al. [30]. Membranes were later stripped and probed sequentially with α-EGFR and α-PTP1B antibodies. Data are presented as the mean of three independent experiments ± SD. * *p*-value < 0.05, ** *p*-value < 0.01 (compared to WT PTP1B). The membrane is representative of three independent experiments.

To assess the intrinsic protein tyrosine phosphatase activity of WT PTP1B and the three mutants, we carried out reverse-phase ultra-fast liquid chromatography (RP-UFLC) using a known tyrosine-phosphorylated peptide substrate of PTP1B (sequence: KGTGYphosphoIKTE) derived from human STAT1 as previously described [28,29]. Compared to WT PTP1B, the intrinsic protein tyrosine phosphatase activity of the three mutants was found to be extremely low; the V184D mutant displayed around 5% residual activity, and the R221G and G259V mutants were almost inactive (Figure 2A).

Similar results were obtained when a classical non-peptidic phosphatase substrate (*p*NPP) known to be dephosphorylated by PTP1B was used (Supplementary Figure S1). These data show that the three PMBCL-associated mutations (V184D, R221G, G259V) that occurred in the active site loops led to PTP1B variants that were strongly impaired in their intrinsic tyrosine phosphatase activity. Our results on the V184D PTP1B mutation concur with activity assays conducted by Gunawardana et al. in extracts of transfected cells that show that this mutant, albeit strongly impaired, retained some residual tyrosine phosphatase activity (~30% activity compared to the WT) [7]. Nonetheless, as these activities were measured in cells extracts, it is thus possible that other PTP enzymes present in the extracts contributed to the overall tyrosine phosphatase activity. The two other PTP1B mutants, R221G and G259V identified in PBMCL, were not analyzed in the Gunawardana et al. study [7]. To further characterize the V184D, R221G, and G259V PTP1B mutants, transfection studies were carried out in HEK293T cells followed by immunoprecipitation and measurement of their intrinsic tyrosine phosphatase activity by RP-UFLC as previously described [29]. In agreement with the results reported above with the recombinant purified proteins, we found that the R221G and G259V mutants displayed no intrinsic protein tyrosine phosphatase activity while the V184D mutant displayed around 5% of the WT activity (Figure 2B). Transfection studies in COS7 cells expressing the EGFR receptor (which is a well-known endogenous substrate of PTP1B) were carried out to analyze the tyrosine phosphatase activity of the mutants as described previously [30]. As shown in Figure 2C, compared to WT PTP1B, the three mutants were much less effective at dephosphorylating EGFR in transfected cells, thus further supporting that the V184D, R221G, and G259V PTP1B mutants have impaired intrinsic tyrosine phosphatase activity in cells. Interestingly, similar results were obtained for the V184D PTP1B mutation in transfection studies where this mutant was shown to poorly dephosphorylate pSTAT6 [7]. However, further studies are needed to ascertain whether the differences in tyrosine phosphorylation due to these PTP1B mutants translate into a significant biological effect in vivo.

As the V184D mutant was active enough for accurate quantitative measurement of its phosphatase activity in this study, we further characterized it. We found that the mutation impacted substrate binding (K_m_ constant), and more importantly, the catalytic constant (k_cat_) gave rise to a mutant with a strongly decreased (8 to 10 times lower compared to the WT) catalytic efficiency (k_cat_/K_m_) (Supplementary Figure S2). This is in agreement with the key role of the WPD-loop in the catalysis and substrate interaction [21,22]. Interestingly, similar results were obtained with a V184G mutant that was found to be poorly active with a catalytic efficiency 10 times lower compared to the WT [22].

**Figure 3:**
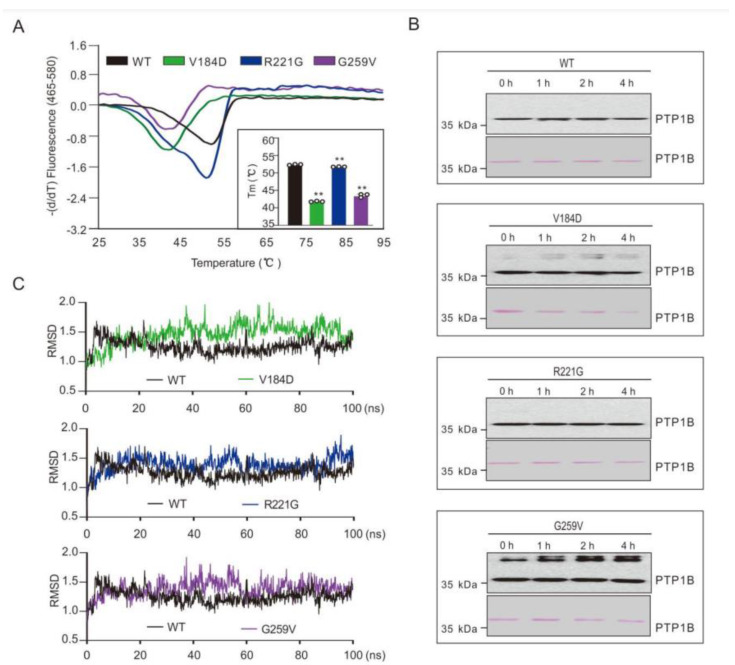
Evaluation of the PTP1B protein stability in the WT and mutants. (**A**) Thermal shift assay of the PTP1B proteins (5 µM) in the presence of 10 X SYPRO in the WT and mutants. T_m_ values were determined from the first-order derivative of the fluorescence as a function of temperature. Data are presented as the mean of three independent experiments ± SD. ** *p*-value < 0.01 (compared to WT PTP1B). (**B**) The PTP1B proteins (10 μg) were incubated at 37 °C; aliquots of the solution were taken at different time points (0, 1, 2, 4 h), separated by SDS-PAGE, and subjected to PTP1B immunodetection following Ponceau staining. The membrane shown is representative of three independent experiments. (**C**) Molecular dynamics simulation of the PTP1B proteins in the WT and mutants was conducted for 100 ns using Amber20 software (PTP1B PDB code: 2HNP). Data were extracted with VMD and presented in the form of RMSD.

To test whether the mutations affected the overall protein stability of V184D, R221G, and G259V, a PTP1B protein (using catalytic domain residues 1–321) thermal shift assay (TSA) was carried out as previously described [31]. As shown in Figure 3A, the V184D and G259V mutants showed strongly decreased thermal stabilities when compared to the WT enzyme (temperature of melting, T_m_, around 42 °C for the two mutants compared to 52 °C for the WT). The R221G mutant showed a very modest decrease (T_m_ = 51 °C). Consistent with these results, the V184D and G259V mutants were also found to be more prone to temperature-dependent protein aggregation as shown in Figure 3B. In addition, determination of the first-order heat inactivation constant (k_inact_) for V184D (k_inactV184D_ = 67.10^−3^ min^−1^) and for WT PTP1B (k_inactWT_ = 21.10^−3^ min^−1^) confirmed that the V184D mutant had a lower intrinsic stability (Supplementary Figure S3). Molecular dynamics (MD) simulations were carried out to further analyze the intrinsic stability of the PTP1B mutants. Interestingly, this approach was used previously for the study of a point mutation of PTP1B Asp181 and in particular, its impact on protein flexibility/stability [32]. The root mean square deviation (RMSD) values for the WT PTP1B backbone Cα atoms fluctuated around 1.3 Å, and the amplitude remained within 1.5 Å, and for the PTP1B mutants, the values were higher, with fluctuations above 1.5 Å and amplitudes close to 2 Å, in particular for the V184D and G259V PTP1B mutants (Figure 3C).

These data further support that, in addition to their strong impact on intrinsic tyrosine phosphatase activity, the V184D, R221G, and G259V mutations also impacted intrinsic protein stability. Interestingly, it has been shown that oncogenic point mutations affecting the catalytic domain of human PTPRT lead to impaired intrinsic enzyme activity and also to decreased intrinsic protein stability of the mutants compared to the WT [33].

**Figure 4:**
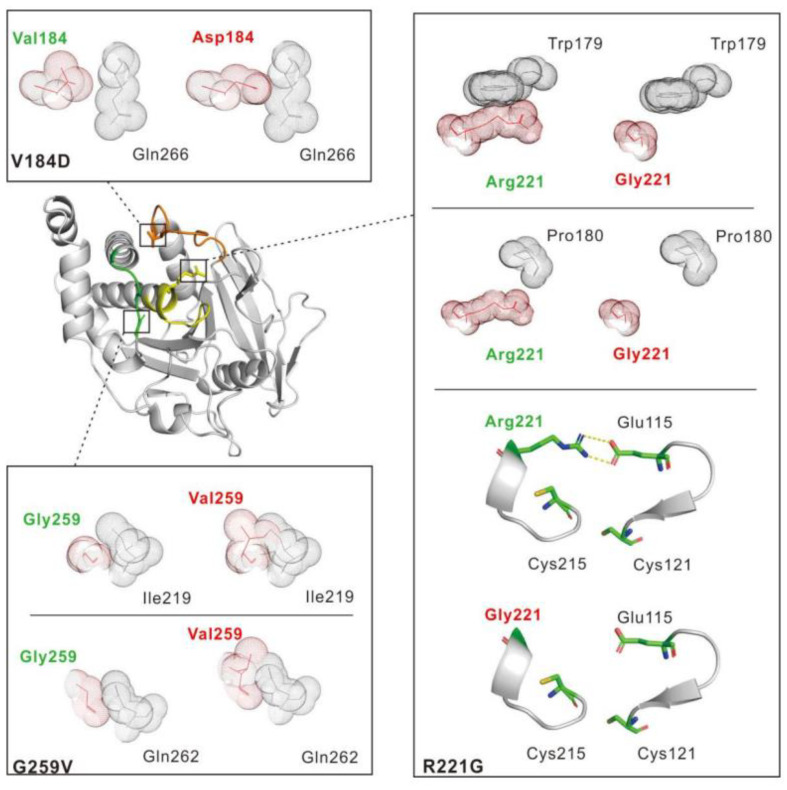
Impact of the V184D, R221G, and G259V mutations on the PTP1B structure. The PTP1B mutants were generated in silico using PyMol software (PTP1B PDB code: 2HNP). The positions of the mutations are shown in the PTP1B global structure. The WPD-Loop (residues 177–188), catalytic P-loop (residues 214–223), and Q-loop (residues 259–263) are shown in orange, yellow, and green, respectively. Close-up views of the V184D, R221G, and G259V mutations are displayed. Van der Waals radii are represented for each atom, and mutated residues are displayed in red. Salt bridges are depicted with yellow dashed lines.

As stated above, the V184D, R221G, and G259V PTP1B mutations identified in PMBCL occurred in three highly conserved adjacent loops (WPD-loop, P-loop, and Q-loop) that contribute to the active site of the enzyme [21] (Figure 4).

**Figure 5:**
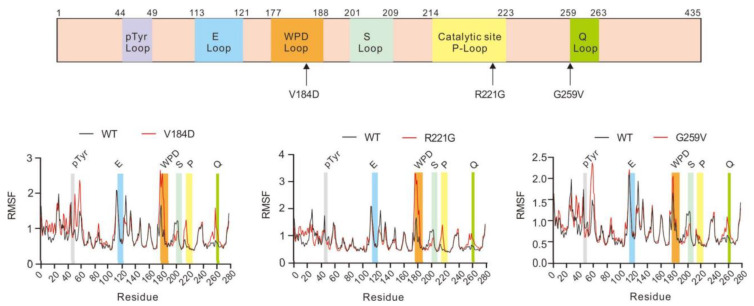
Molecular dynamic simulations of PTP1B in the WT and mutants and the impact on the active site loops. Upper panel: Schematic representation of the different key loops of PTP1B (residues 1–435; pTyr-loop is shown in purple, E-loop in blue, WPD-loop in orange, S-loop in light green, catalytic P-loop in yellow, and Q-loop in green). Lower panel: Molecular dynamics simulations of the PTP1B proteins (100 ns simulations) in the WT and mutants using Amber20 software. Data were extracted with VMD and presented as RMSF. Differences between PTP1B in the WT and mutants were highlighted according to the loop color scheme defined in the upper panel.

Val184 is a highly conserved WPD-loop residue in PTP enzymes. The WPD-loop is a mobile loop that also contains the key catalytic acid/base Asp181 residue. Upon substrate binding, the WPD-loop closes around the active site so that the catalytic Asp181 is correctly positioned to participate in catalysis [21,22,34]. As shown in Figure 4, replacement of a Val residue with Asp leads to a steric clash with the residue Gln266, a residue that flanks the catalytically important Q-loop [21]. This clash may impact the flexibility and motion of the WPD-loop, thus altering the activity of the enzyme. MD analysis showed a significant increase in the root mean square fluctuations (RMSF) of the Cα of the WPD-loop residues (residues 177–188) in the V184D mutant compared to the WT (with ΔRMSF close to 1 Å). The RMSF values reflect the mobility of a given residue around its mean position and thus provide important information about the flexibility and stability of the system [32]. Thus, these data clearly indicate that the V184D mutation strongly impacts the flexibility and the dynamics of the WPD-loop. Interestingly, it is now well-known that the conformational dynamics of the WPD-loop are key for enzyme activity, notably through the positioning of catalytically important WPD-loop residues, in particular Asp181 [22,24]. In addition, MD analysis indicated that the V184D mutation, by impacting the structure/motion of the WPD-loop, also modifies the flexibility of other active site loops, notably the Q-loop (residues 259–263) and the P-loop (residues 214–223) (Figure 5).

This is in agreement with the fact that these loops are adjacent to and form the catalytic site [21]. In addition, as stated above, the V184D mutation induced a steric clash with the Gln266 residue that flanks the Q-loop. The latter contains the very important Gln262 residue whose flexibility and correct positioning in the active site are key for catalysis in conjunction with the Asp181 residue of the WPD-loop [21,22,35]. It is not surprising that a mutation impacting the structure/flexibility of one active-site loop, such as the WPD-loop, also modifies the structure/flexibility of the neighboring loop [24]. Interestingly, it is now well known that the WPD-loop and other active site loops (P-loop and Q-loop notably) are part of network that also includes dispersed residues that contribute to enzyme activity through modulation of loop motions (in particular the motion of the WPD-loop) [22,23,24]. In agreement with this, the MD dynamics of the V184D mutant showed significant RMSF differences with WT PTP1B in terms of the Q-loop, the P-loop, and also residues 52–56 and residues 191–193 (Figure 5). Interestingly these residues are known to be “allosterically” connected to the WPD-loop motion and enzyme activity [22,25].

The Arg221 residue is also a highly conserved residue that belongs to the P-loop motif (Figure 5). This residue plays a key structural and catalytic role, notably through interaction with the phosphate group of the substrate during catalysis and also through ionic bonds with the Glu115 residue of the E-loop (residues 113–121) [21]. In addition, interactions of Arg221 with the WPD-loop Trp179 and Pro180 residues are important in particular for closure of the WPD-loop during catalysis [22]. The R221G mutation introduces a small non-charged glycine residue that, contrary to the Arg residue, cannot form ionic or H-bonds and cannot interact with the WPD-loop Trp179 and Pro180 residues (Figure 4). These radical changes likely explain why the R221G PTP1B mutant is devoid of tyrosine phosphatase activity. As stated above, the MD dynamics analyses of the R221G mutant confirmed that the mutation impacts the flexibility of the P-loop, which could be due, at least in part, to the lack of a side-chain of the Gly residue at position 221, thereby providing more conformational flexibility to adjacent P-loop residues (Figure 5). More importantly and in agreement with the points discussed above, the mutation of the Arg221 residue of the P-loop not only impacts the flexibility of this loop but also give rise to significant changes in the flexibility of the WPD-loop (with ΔRMSF around 1 Å) and more modestly of the Q-loop (ΔRMSF around 0.5 Å) (Figure 5). This is consistent with the fact that Arg221 interacts directly with WPD-loop residues (Trp179 and Pro180) [22].

The Gly259 residue is part of the Q-loop (residues 259–263) (Figure 5). Due to the absence of a side chain, the Gly259 residue has been shown to allow easy access to the substrate in the active site. Interestingly, the mutation of Gly259 into a Gln residue has been shown to impair PTP1B activity by causing steric hindrance but also by impacting the flexibility and correct positioning of the Q-loop Gln262 residue in the active site during catalysis [35]. As shown in Figure 4, the G259V PTP1B mutation identified in PMBCL patients leads to a significant steric clash with the key catalytic residue Gln262, which may prevent the Gln262 residue from adopting a correct position during catalysis [21,23,35]. MD analyses confirmed that the mutation impacted the flexibility of the Q-loop (with ΔRMSF around 0.5 Å) but also of the WPD-loop and certain residues of the PTP1B allosteric network (residues 52–56 and residues 191–193) (Figure 5). These data are in agreement with previous results showing that mutation of the Gly259 residue into a bulkier Gln residue leads to altered flexibility of the key catalytic Gln262 residue and subsequently to a poorly active PTP1B mutant enzyme [35].

Taken together, our results provide molecular, structural, and enzymatic evidence that the three P1PT1B mutants (V184D, R221G, and G259V) identified in human primary mediastinal B cell lymphoma are strongly impaired in their protein tyrosine phosphatase activity. This enzymatic impairment may contribute to aberrant phosphotyrosine cell signaling as suggested previously by Gunawardna et al. [7]. Our data further underline the possible pathogenic role of oncogenic mutations that affect active-site loops in PTP enzymes [36].

## 3. Materials and Methods

### 3.1. Materials

*p*-nitrophenyl phosphate (*p*NPP), HClO_4_, dithiothreitol (DTT), sodium fluoride (NaF), sodium orthovanadate (Na_3_VO_4_), isopropyl β-D-thiogalactoside (IPTG), Triton X-100, lysozyme, His-select nickel resin, and SYPRO were obtained from Sigma–Aldrich (Saint Quentin-Fallavier, France). Protease inhibitors were purchased from Roche (Meylan, France). PD-10 columns were obtained from Cytiva (Velizy, France). The α-EGFR antibody (SAB4300352) and Protein G plus-agarose beads (SC-2002) were obtained from Santa Cruz Biotechnology (Heidelberg, Germany). The α-PTP1B antibody (FG6-1G) was purchased from Merck (Saint Quentin-Fallavier, France), and the α-pan-phospho-tyrosine antibody (9411) was obtained from Cell Signaling (Leiden, The Netherlands).

### 3.2. Cell Culture

HEK 293T and COS7 cells were cultured in DMEM (Gibco, Les Ulis, France) supplemented with 10% fetal bovine serum (Sigma, Saint-Quentin Fallavier, France) at 37 °C under 5% CO_2_.

### 3.3. Generation of PTP1B Mutants

Mutations V184D, R221G, and G259V of PTP1B were generated in pET28-PTP1B (residues 1–321) and pCMV flag-PTP1B (full-length) plasmids using the Agilent QuikChange XL site-directed mutagenesis kit (TX, USA) with the following mutagenesis primers. The presence of the mutations was then verified by DNA sequencing (Eurofins, Ivry/Seine, France).

V184D-For: GGCCTGACTTTGGAGACCCTGAATCACACCAG

V184D-Rev: CTGGTGTGATTCAGGGTCTCCAAAGTCAGGCC

R221G-For: GTGCAGGCATCGGCGGGTCTGGAACCTTC

R221G-Rev: GAAGGTTCCAGACCCGCCGATGCCTGCAC

G259V-For: GGAAGTTTCGGATGGTGCTGATCCAGACAGC

G259V-Rev: GCTGTCTGGATCAGCACCATCCGAAACTTCC

### 3.4. Expression and Purification of Recombinant PTP1B Enzymes

The pET28 WT and mutant PTP1B plasmids were transformed into BL21 HI-Control™ (DE3) *E. coli*. Bacteria were cultured at 37 °C under agitation until reaching an OD of 0.6 and then grown overnight at 16 °C in the presence of 0.5 mM IPTG. Cells were harvested by centrifugation and resuspended in lysis buffer (20 mM Tris HCl, 100 mM NaCl, 0.5% Triton X-100, 1 mg/mL lysozyme and protease inhibitor, pH 7.5). The lysate was sonicated (10 s ON, 20 s OFF, 10 min, 20% power) on ice and pelleted (15,000× *g*, 30 min, 4 °C). The supernatant was incubated with His-select nickel resin in the presence of 10 mM imidazole for 2 h at 4 °C under agitation. Beads were then poured into a column, washed with washing buffer (20 mM Tris-HCl, 100 mM NaCl, 20 mM imidazole, pH 7.5), and the enzyme was eluted with elution buffer (20 mM Tris-HCl, 100 mM NaCl, 300 mM imidazole, pH 7.5). Protein elution was controlled by Bradford assay, and eluted samples were incubated with 10 mM DTT on ice for 20 min. Purified enzyme was buffer exchanged to 20 mM Tris-HCl, 100 mM NaCl, pH 7.5 using a PD-10 column. Protein concentration and purity were assessed by Bradford reagent and SDS-PAGE. Proteins were stored at −80 °C until use.

### 3.5. Determination of Recombinant PTP1B Activity Using the FAM-Conjugated Tyrosine-Phosphorylated STAT1 (pSTAT1) Peptide by RP-UFLC

The tyrosine phosphatase activity of the enzymes was measured by RP-UFLC using a FAM-conjugated peptide derived from the sequence of human STAT1 where tyrosine 701 was phosphorylated (KGTGY_701_IKTE, Proteogenix, Schiltigheim, France) as previously described [29]. Briefly, samples containing the PTP1B enzymes were incubated with FAM-conjugated pSTAT1 peptide in 100 mM sodium acetate buffer, 1 mM DTT, pH 6, for 30 min at 37 °C (total volume of 50 μL). The reaction was stopped with the addition of 50 μL HClO_4_ (15%, *v*/*v*), and samples were injected into a Shim-pack XR ODS column (100 mm, 2 mm, 2.2 μm) (Shimadzu, Noisiel, France) at 40 °C. The mobile phase used for the separation consisted of two eluents: solvent A was water with 0.12% trifluoacetic acid (TFA), and solvent B was acetonitrile with 0.12% TFA. Compounds were separated by an isocratic flow (80% A/20% B) rate of 0.6 mL/min. The pSTAT1 peptide and its dephosphorylated form (product) were monitored by fluorescence emission (λ = 530 nm) after excitation at λ = 485 nm and were quantified by integration of the peak absorbance area.

### 3.6. PTP1B Transfection, Immunoprecipitation, and Tyrosine Phosphorylation Immunodetection in HEK 293T and COS7 Cells

HEK 293T and COS7 cells were seeded at 100,000 cells/cm^2^ in a 100 cm^2^ Petri dish and directly transfected using a solution containing 3 µg of the WT’s or mutants’ pCMV flag-PTP1B and 6 µL metafectene (Biontex, Munchen, Germany) according to the manufacturer’s instructions. Cells were then put back in the incubator at 37 °C, 5% CO_2_, for 48 h.

For PTP1B immunoprecipitation, transfected HEK 293T cells were later washed with PBS, harvested, and lysed in lysis buffer (PBS 1X, 1% Triton X-100 and protease inhibitor) at 4 °C for 30 min under agitation. Cell lysates were centrifuged at 15,000× *g* for 10 min at 4 °C, and the supernatants (whole-cell extracts) were retained. Transfected PTP1B was immunoprecipitated by incubating 1 mg of whole-cell extracts in the presence of 1 µg of PTP1B antibody overnight at 4 °C under agitation. Samples were then rocked for 1 h at 4 °C in the presence of 30 μL protein G–agarose beads. Immunobeads were harvested by centrifugation, washed three times with lysis buffer (PBS, 1% Triton X-100), and split in two. Half of the beads were incubated with 50 µM FAM–phosphorylated STAT1 peptide in sodium acetate buffer (100 mM, 1 mM DTT, pH 6) at 37 °C for 2 h (total volume of 50 μL) to determine PTP1B residual activity by RP-UFLC as described previously [28]. The second part of the immunobeads was incubated with non-reducing Laemmli sample buffer and heated at 100 °C for 10 min. Samples were separated by SDS-PAGE, transferred onto nitrocellulose membrane, and analyzed by Western blot using the α-PTP1B antibody.

COS7 cells transfected with the WT and the different PTP1B mutants were used to evaluate the tyrosine phosphorylation of EGFR as previously described by Flint et al. [30]. Briefly, transfected COS7 cells were washed with PBS, harvested, and lysed in lysis buffer (PBS 1X, 1% Triton X-100, 1 mM Na_3_VO_4_, 1 mM NaF and protease inhibitors) at 4 °C for 30 min under agitation. Cell lysates were centrifuged at 15,000× *g* for 10 min at 4 °C, and the supernatants (whole-cell extracts) were retained. Samples were separated by SDS-PAGE and transferred onto a nitrocellulose membrane. EGFR tyrosine phosphorylation was then assessed by Western blot using an anti-phospho-tyrosine antibody as described by Flint et al. [30]. Membranes were later stripped and re-probed sequentially with α-EGFR and α-PTP1B antibodies.

### 3.7. Determination of PTP1B Intrinsic Stability by Thermal Shift Assay (TSA)

TSA was performed to monitor the thermal intrinsic stability of PTP1B proteins. Briefly, the PTP1B proteins (5 μM) of the WT and mutants were incubated in the presence of 10X SYPRO orange dye in a total volume of 10 μL buffer (25 mM Tris, 100 mM NaCl, pH 7.5) in 384-well opaque PCR plates (Roche, Meylan, France). The plates were then centrifuged (1300 rpm, 4 °C), and real-time analysis of the thermal stability of the proteins was performed by fluorescence reading (excitation: 465 nm, emission: 580 nm) on a Light Cycler 480 (Roche, Meylan, France). The program was as follows: (1) 15 s incubation at 20 °C; (2) temperature rise from 20 °C to 95 °C at an increment of 0.05 °C/s and 12 readings/degree; and (3) return to 20 °C for 15 s. The obtained fluorescence data were analyzed and quantified using a LightCycler 480 SW 1.5. Tm values were measured by determining the minimum of the first derivative of fluorescence emission as a function of temperature (dFluo/dT).

### 3.8. Evaluation of Protein Stability/Aggregation by Western Blot

The PTP1B proteins (10 μg) of the WT and mutants were incubated at 37 °C in a total volume of 50 μL (25 mM Tris, 100 mM NaCl, pH 7.5). At different time points (0, 1, 2, 4 h), 10 µL of the reaction mixture was taken, and samples were separated by SDS-PAGE and transferred onto a nitrocellulose membrane. Ponceau staining was conducted on the membrane prior to incubation with the α-PTP1B antibody.

### 3.9. Molecular Dynamics of the WT PTP1B and Mutant PTP1B

The PTP1B WT (PDB code: 2HNP) and its three mutants generated by PyMol (version 2.5, academic license) with the fewest constraints were simulated using Amber20 software and the Charmm36m force field [37]. The biomolecules were solvated with water molecules, and a rectangular system with an edge distance of 10 Å was selected. Na^+^ ions were also added to neutralize the system using the Monte Carlo method. Periodic boundary conditions were then applied according to the shape and size of the system. The Particle Mesh Ewald (PME) FFT mesh information was determined, and then minimization was performed to eliminate steric clashes. The temperature was increased from 0 K to 303.15 K for 5 ns at a constant volume and temperature. After equilibrium was reached, molecular dynamics was performed for 100 ns at a constant pressure and temperature of 1 atm and 303.15 K, respectively. Trajectories every 0.1 ns and an output file were recorded. Visual Molecular Dynamics (VMD) software was used to load the visualized trajectory file to analyze the root mean square deviation (RMSD) of the protein backbone atoms as well as the root mean square fluctuation (RMSF) of the residues [38].

### 3.10. Statistical Analysis

All data were reported as the mean ± standard deviation (S.D.) of three independent experiments. Quantification of the Western blot signal was carried out using ImageJ software (National Institutes of Health, USA). Data were analyzed by one-way analysis of variance (ANOVA) using GraphPad Prism version 8.0.0. A threshold of *p* < 0.05 was used to consider differences statistically significant. (* *p* ≤ 0.05, ** *p* ≤ 0.01).

## Supplementary Materials

The following supporting information can be downloaded at: https://www.mdpi.com/article/10.3390/ijms23137060/s1. References [28,39] are cited in Supplementary Material.

## Abbreviations

PTP: protein tyrosine phosphatase; PTP1B: protein tyrosine phosphatase 1B; PMBCL: primary mediastinal B cell lymphoma; RP-UFLC: reverse-phase ultra-fast liquid chromatography; TSA: thermal shift assay; MD: molecular dynamics.
