# Supplementary material for: Biochemical, Enzymatic, and Computational Characterization of Recurrent Somatic Mutations of the Human Protein Tyrosine Phosphatase PTP1B in Primary Mediastinal B Cell Lymphoma
Source: Int J Mol Sci. 2022 Jun 24;23(13):7060. doi: 10.3390/ijms23137060 (PMC9266312; doi:10.3390/ijms23137060)
Supplement: Supplementary file 1 [file ijms-23-07060-s001.zip › ijms-1772037-supplementary.pdf]

Supplementary

Biochemical, enzymatic and computational characterization of recurrent somatic mutations of the human protein tyrosine phosphatase PTP1B in primary mediastinal B cell lymphoma

Rongxing Liu[1], Yujie Sun[2], Jérémy Berthelet[3], Linh-Chi Bui[1], Ximing Xu[2], Mireille Viguier[1], Jean-Marie Dupret[1], Frédérique Deshayes[1] and Fernando Rodrigues Lima[1,*]

[1] Université Paris Cité, CNRS, Unité de Biologie Fonctionnelle et Adaptative, F-75013, Paris, France
[2] School of Medicine and Pharmacy, Ocean University of China, Qingdao, 266071, Shandong, China
[3] Université Paris Cité, CNRS, Centre d'Epigénétique et Destin Cellulaire, F-75013, Paris, France

* Correspondence: Fernando.rodrigues-lima@u-paris.fr (F Rodrigues Lima)

Running title: Effects of lymphoma associated mutations on PTP1B

SUPPLEMENTARY MATERIAL

1. Determination of PTP1B activity using *p*NPP assay

The tyrosine phosphatase activity of WT and mutants PTP1B proteins were measured by *p*NPP assay as previously described (Nian *et al.*, 2019). Briefly, samples containing PTP1B (20 nM) were incubated with *p*NPP in 100 mM sodium acetate buffer, 1 mM DTT, pH 6 (total volume of 200 µl). The formation of the product (*p*-nitrophenol) was followed by continuous measurement of the absorbance at 405 nm for 10 minutes at 37°C using thermostatic microplate reader (BioTek, France).

2. Determination of WT and V184D PTP1B kinetic parameters

Kinetic parameters of WT and V184D PTP1B were assessed using *p*NPP and RP-UFLC assays. For *p*NPP assay, WT and V184D PTP1B proteins (20 nM) were mixed with increasing concentrations of *p*NPP (ranging from 1.25 to 20 mM) and PTP1B activity was determined as described before (Nian *et al.*, 2019). For RP-UFLC assay, WT and V184D PTP1B proteins (5 nM) were incubated with increasing concentrations of pSTAT1 peptide (ranging from 25 to 400 µM) for 30 minutes at 37°C and PTP1B activity was determined as described before (Duval *et al.*, 2015). The apparent kinetic parameters V_{max}, K_m and k_{cat} were obtained by non-linear curve fitting against the Michaelis–Menten equation using Prism 8.0.0 software.

3. Determination of first-order heat inactivation constants for WT and V184D PTP1B

WT and V184D PTP1B (20 nM) were incubated at 37°C. At different time points (0, 3, 6, 9, 12 minutes), an aliquot of the solution was taken and mixed with *p*NPP (5 mM) in a total volume of 200 µl sodium acetate buffer (100 mM, 1 mM DTT, pH 6). Natural logarithm of PTP1B residual activity tested by *p*NPP assay was then plotted as a function of time using Prism 8.0.0 software.

Supplementary legend figures

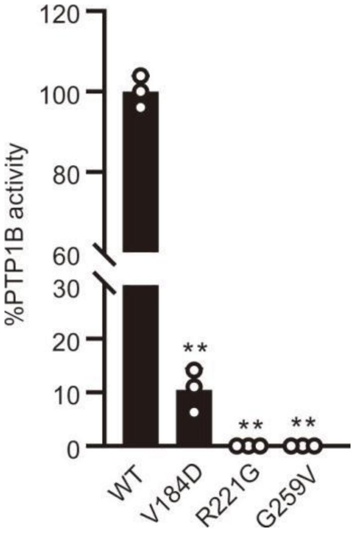

Figure S1: Determination of WT and mutants PTP1B activity using *p*NPP assay

WT or mutants PTP1B enzyme (20 nM) activity was determinated using *p*NPP assay. Data are represented as the mean of three independent experiments +/- SD. ** p-value < 0.01 (compared to WT PTP1B).

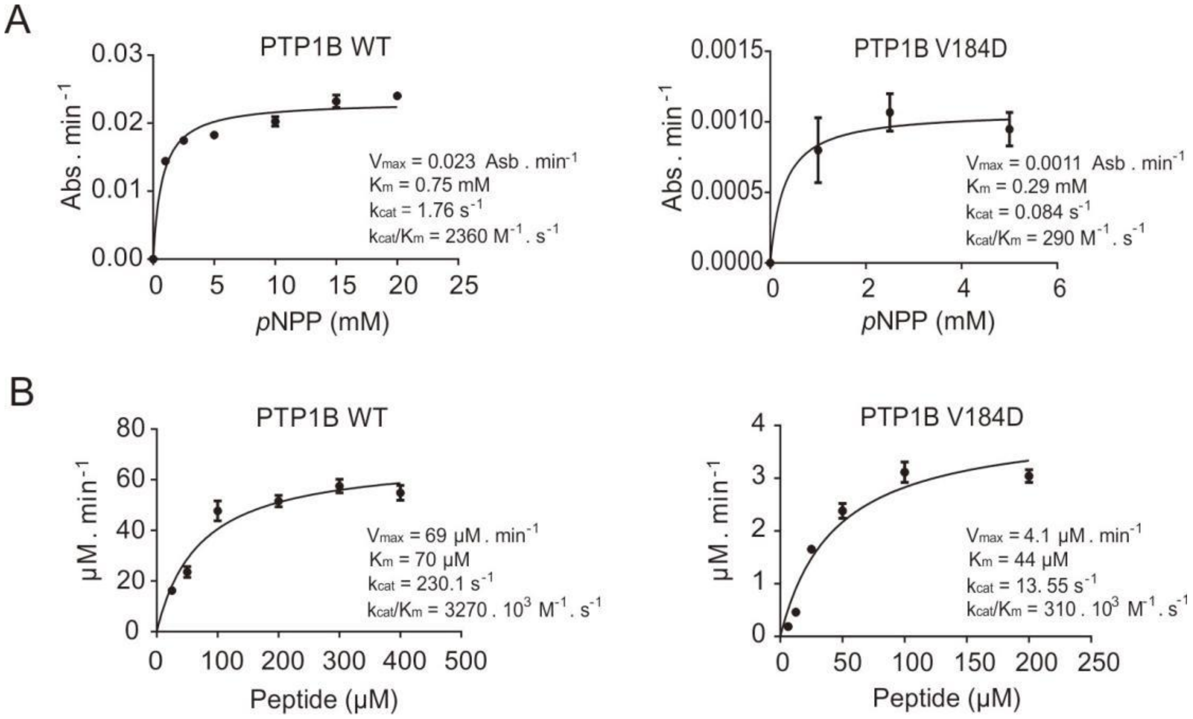

Figure S2: Determination of WT and V184D PTP1B kinetic parameters

(A/B) WT or mutants PTP1B enzyme (20 nM) activity was assessed using different *p*NPP concentrations (ranging from 1.25 mM to 20 mM). The apparent kinetic parameters V_{max}, K_m and k_{cat} were obtained by non-linear curve fitting against the Michaelis–Menten equation. Data are represented as the mean of three independent experiments +/- SD.

(C/D) WT or mutants PTP1B enzyme (5 nM) were incubated in the presence of different concentrations of pSTAT1 peptide (ranging from 25 to 400 µM) for 30 minutes at 37°C. PTP1B activity was then assessed by RP-UFLC. The apparent kinetic parameters V_{max}, K_m and k_{cat} were obtained by non-linear curve fitting against the Michaelis–Menten equation. Data are represented as the mean of three independent experiments +/- SD.

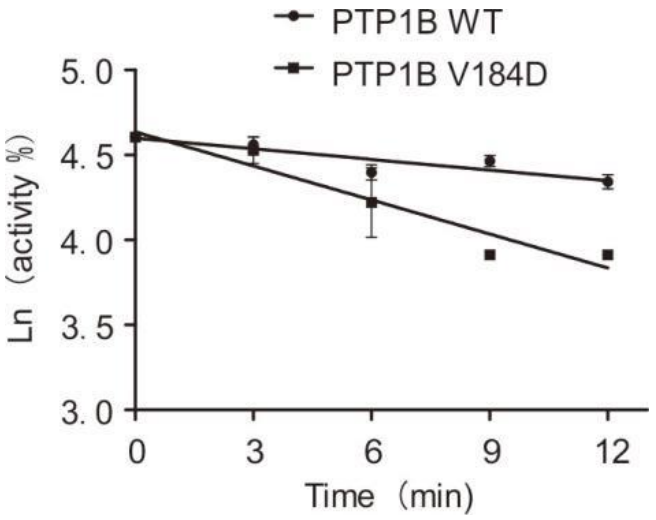

Figure S3: Determination of first-order heat inactivation constant of WT and V184D PTP1B

WT and V184D PTP1B (20 nM) were incubated at 37°C. At different time points (0, 3, 6, 9, 12 minutes), an aliquot of the solution was taken and PTP1B residual activity was assessed using *p*NPP assay. Natural logarithm of PTP1B residual activity was then plotted as a function of time.

REFERENCES

1. Nian, Q.; Berthelet, J.; Zhang, W.; Bui, L.C.; Liu, R.; Xu, X.; Duval, R.; Ganesan, S.; Leger, T.; Chomienne, C.; et al. T-Cell Protein Tyrosine Phosphatase Is Irreversibly Inhibited by Etoposide-Quinone, a Reactive Metabolite of the Chemotherapy Drug Etoposide. *Mol. Pharmacol*. **2019,** 96, 297–306. https://doi.org/10.1124/mol.119.116319.
2. Duval, R.; Bui, L.C.; Berthelet, J.; Dairou, J.; Mathieu, C.; Guidez, F.; Dupret, J.M.; Cools, J.; Chomienne, C.; Rodrigues-Lima, F. A RP-UFLC Assay for Protein Tyrosine Phosphatases: Focus on Protein Tyrosine Phosphatase Non-Receptor Type 2 (PTPN2). *Sci. Rep*. **2015**, *5*, 10750. https://doi.org/10.1038/srep10750.
